# Detection of Epstein-Barr virus (EBV) in human lymphoma tissue by a novel microbial detection array

**DOI:** 10.1186/s40364-014-0024-x

**Published:** 2014-12-05

**Authors:** Joseph Tellez, Crystal Jaing, Jun Wang, Ralph Green, Mingyi Chen

**Affiliations:** Deptartment of Pathology and Laboratory Medicine, University of California, Davis Medical Center, PATH Bldg. 4400V Street, Sacramento, CA 95817 USA; Applied Genomics, Biosciences and Biotechnology Division, Lawrence Livermore National Laboratory, Livermore, CA 94551 USA; Department of Pathology and Laboratory Medicine, Loma Linda University Medical Center, Loma Linda, CA 92354 USA

**Keywords:** Epstein-Barr virus (EBV), Lymphoma, Post-transplant lymphoproliferative disorder (PTLD), Lawrence Livermore Microbial Detection Array (LLMDA)

## Abstract

**Background:**

Infectious agents are estimated to play a causative role in approximately 20% of cancers worldwide. Viruses, notably the Epstein-Barr virus (EBV), are associated with 10-15% of B-cell lymphomas and are found at a higher frequency in immunosuppressed patients. In this study, we screened human lymphoma tissues using a novel Lawrence Livermore Microbial Detection Array (LLMDA), a comprehensive detection system that contains probes for all sequenced viruses and bacteria. This technology has been applied to identify pathogen-associated diseases.

**Results:**

We evaluated samples from 58 cases with various lymphoid tissue disorders using LLMDA. These included 30 B-cell lymphomas (9 indolent and 21 aggressive type), 2 T-cell lymphomas and 2 NK/T cell lymphomas, 4 plasmacytomas as well as 8 specimens of benign lymphoid tissue. Five of 21 high-grade B-cell lymphomas were positive for Epstein-Barr virus-encoded small RNA (EBER+), while all the indolent B-cell lymphomas were EBER-. Similarly, both NK/T cell lymphomas were EBER+, and the benign tissues were EBER-. We also screened 10 cases of post-transplant lymphoproliferative disorder (PTLD). Five of these cases (4 B-cell lymphomas and 1 NK/T cell lymphoma) were EBER+, and the remaining five cases were EBER-.

**Conclusions:**

We have confirmed the reliability of the LLMDA methods by detecting EBV in EBV-positive lymphomas while observing no false-positive results in EBV-negative lymphomas. The LLMDA technique provides a sensitive and alternative method for identifying known viral pathogen associated with tumors and may prove useful for future clinical identification of novel cancer-associated viral pathogens.

## Background

Infection associated cancers are on the rise worldwide due to shifting pathogen habitats, a more interconnected world and most importantly an aging population with a longer life-expectancy [1,2]. As humans age, the immune systems become dysregulated due to immunosenescence leaving them more susceptible to infection (comorbidities and environmental exposures) [3-5]. It is estimated that at least 20% of the global cancer incidence is caused by infectious agents, with 10-15% of those caused by viruses [6]. Over the past three decades, research has linked a number of cancers to infectious agents including viruses (Epstein-Barr virus (EBV), human papilloma virus (HPV), hepatitis B, human T-lymphotropic retrovirus, Kaposi’s sarcoma-associated herpesvirus (KSHV), and Merkel cell virus), bacteria (Helicobacter pylori), and parasites (Schistosoma haematobium, Clonorchis sinesis) [7]. Over 90% of the world’s population is infected with EBV [8]. The organ transplant patients are prone to viral infections due to immunosuppression which leaving them susceptible to post-transplant lymphoproliferative disorder (PTLD) [9]. Immunocompetent individuals control EBV infection with EBV-specific cytotoxic T lymphocytes (CTLs), while immunosuppressed transplant patients lack CTLs and allow EBV propagation that may lead to PTLD [8].

EBV, or human herpesvirus 4, is one of the most common viruses affecting humans. EBV is an episomal, double-stranded DNA virus that was discovered in a Burkitt lymphoma cell line by Epstein et al. [10-12]. EBV’s promiscuous tropism permits infection of a number of different cell types including B-cells, T-cells, NK-cells and epithelial cells [13-15]. Subsequently, EBV was found to be associated with various human malignancies, including nasopharyngeal carcinomas, PTLD, AIDS-associated lymphomas, T cell lymphomas, NK cell lymphomas and Hodgkin’s disease [11]. Importantly, EBV has been shown to induce B cell transformation [11]. EBV-positive lymphomas can be divided into those occurring in immunodeficient individuals, which are virally driven lymphomas, such as PTLD and HIV-associated plasmablastic lymphoma (PBL), and those occurring in immunocompetent individuals. The latter group includes endemic and sporadic Burkitt lymphoma, and some T-and NK-cell malignancies. In the malignancies occurring in immunocompetent individuals, EBV is a cofactor rather than the driving influence [10]. EBV likely exists as an episome with multiple copies in the host cell, making it easier to detect than viruses with one or few genomic copies per cell [12]. EBV episomes have been employed to determine the association of the virus with various aggressive types of lymphomas, indicating that it is likely involved in tumor progression but not tumor initiation, as might be assumed if present in indolent tumors [11].

While it is clear that EBV contributes to the progression of B-cell lymphoproliferative disease in immunosuppressed patients, its role in lymphomagenesis is less clear in immunocompetent individuals [11]. However, the presence of viral genomes in these lymphomas offers interventional targets and several approaches currently under evaluation which include adoptive immunotherapy, interferon, and small molecule targeting strategies in tumor virus biology [7]. A sensitive and comprehensive pathogen detection technology is critical to understand microbial profiles associated with lymphomagenesis and their contribution to the progression to high-grade lymphomas [16]. Sequencing produces the most comprehensive and unbiased information for microbial detection and discovery when analyzing nucleic acids from uncharacterized samples, but high-throughput sequencing is time-consuming and expensive [17]. PCR is a cheap, fast and sensitive option, but it lacks the ability to detect the existence of large numbers of organisms simultaneously [18]. Microarray technology offers a reliable and sensitive alternative to sequencing and PCR when analyzing or screening tissue samples from patients with known or suspected pathological conditions [18]. Moreover, microarrays are cheaper and faster than sequencing and permit detection of multiple microbes in the same sample [19]. In this study, we used the Lawrence Livermore Microbial Detection Array (LLMDA), which is a pan-microbial detection array capable of detecting all sequenced virus, bacteria and plasmids, which uses a unique statistical method, Composite Likelihood Maximization Method for identifying multiple organisms in complex mixtures [18-21]. The family-specific probes selected were conserved enough to detect all known viral and bacterial organisms while containing sufficient sequence variation for the detection of divergent species with homology to sequenced organisms [22,23]. Our previous studies have demonstrated the potential usage of the LLMDA for detection of a broad spectrum of pathogens in a diverse set of clinical samples. We found that the microarray technique can detect both DNA and RNA viruses that are present in the same sample, as well as differentiate between different virus subtypes [18-20].

In this study we demonstrated the reliability of the LLMDA technique in the detection of pathogen-associated lymphoproliferative diseases. The LLMDA accurately identified EBV in EBV-positive samples and did not register false positive results in EBV-negative samples. We detected EBV in 5 out of 10 PTLD samples and showed that EBV-positivity is usually correlated with aggressive stages of lymphomas. We also demonstrated that the LLMDA can also be applied to detect EBV in Formalin Fixed Paraffin Embedded (FFPE) tissues which widens our sample pool and improves selection of relevant samples. Our results demonstrated that the LLMDA is a powerful tool for analyzing and detecting known microbial pathogens in lymphoproliferative disorders and potentially can be used as a diagnostic tool or to provide a basis for potential targeted and effective therapy regimens for these disorders.

## Results

### Detection of EBV in FFPE lymphoma samples

**Figure 1:**
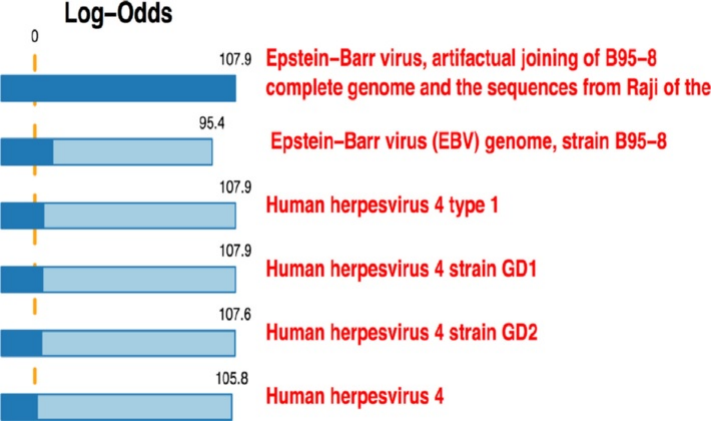
**LLMDA’s detection of EBV in FFPE malignant lymphoma tissue from a 10 μm section with a 150 mm** ^**2**^ **surface area.** The Epstein–Barr virus (EBV), also called human herpesvirus 4 (HHV-4). Array data was analyzed using the Composite Likelihood Maximization Method developed at LLNL. The lighter and darker-colored portions of the bars represent the unconditional and conditional log-odds scores, respectively. The conditional log-odds scores shows the contribution from a target that cannot be explained by another, more likely target above it, while the unconditional score illustrates that some very similar targets share a number of probes. 20 out of the 24 probes specific for EBV were detected on the LLMDA. The log odds score for EBV B95-8 genome is 107.9.

**Figure 2:**
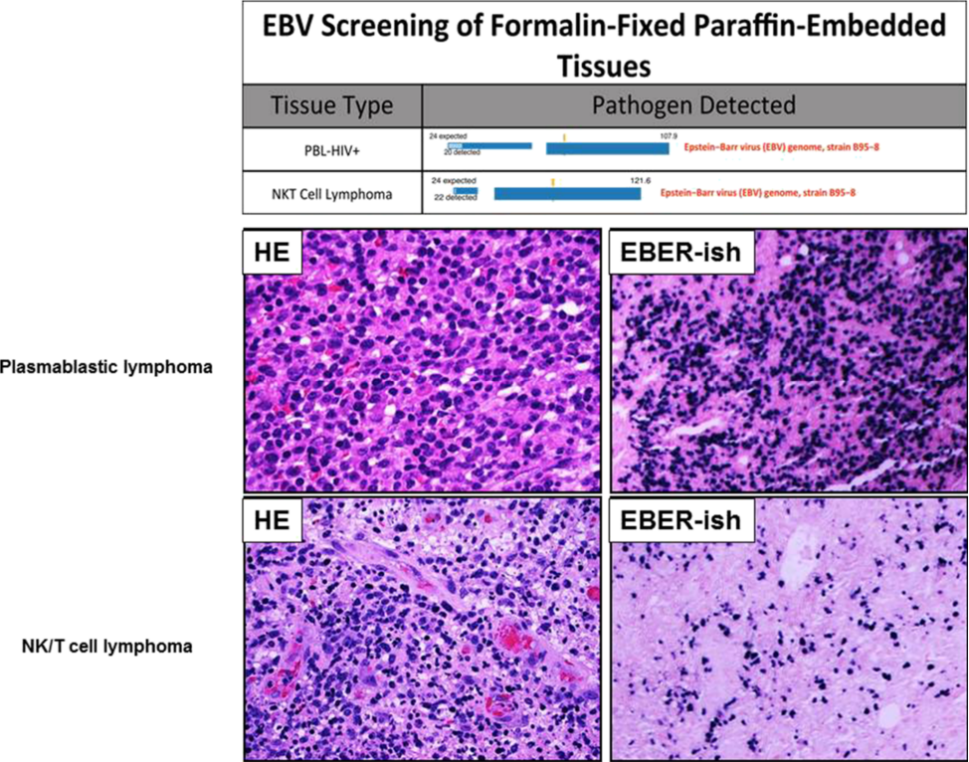
**Representative cases EBV positive malignant lymphoma detected by LLMDA in FFPE tissues.** The assay can detect the very high viral volume in plasmablastic lymphoma as well as relatively low viral volume in T-cell lymphoma.

**Figure 3:**
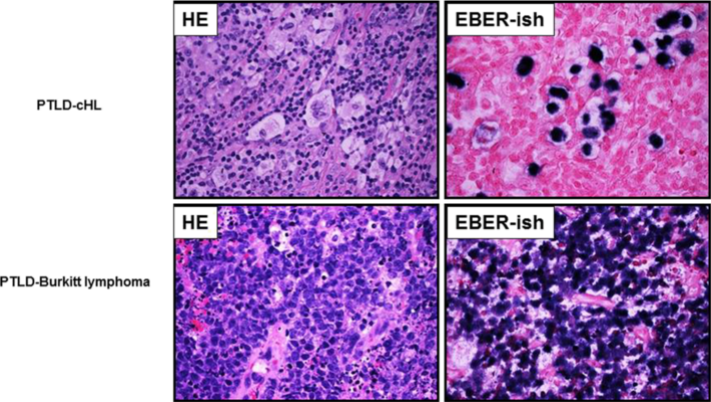
**Validation of the reliability of the LLMDA method for screening of monomorphic and classical Hodgkin lymphoma types of PTLD lymphoma specimens.** The LLMDA accurately detected the presence or absence of EBV in every case: 5 EBV-positive cases and 5-EBV-negative cases.

Though frozen tissue samples are ideal for nucleic acid analysis, obtaining the requisite number of fresh frozen samples can be challenging and is not always possible. Therefore, we have explored the use of FFPE samples for pathogen analysis. FFPE samples are in available abundant supply since the advent and widespread use of tissue banking, making them a good source for obtaining and selecting adequate sample numbers for retrospective analysis. The LLMDA detected EBV in two of two EBV-positive FFPE samples with 10 μm sections. The LLMDA was able to detect EBV in all EBV-positive samples from as little as 1 μm sections; and no false positives were registered (Figure 1 and Table 1). The Epstein–Barr virus (EBV), also called human herpesvirus 4 (HHV-4), is a virus of the herpes family. A case of NK/T cell lymphoma was shown in Figure 2. The top matched genome sequence on the LLMDA is EBV B95-8. The human herpesvirus 4 type 1 and GD1 also have the same log odds score, a composite score to predict the probability of detection. 20 probes out of 26 expected to be specific to EBV were detected, or 83% of EBV probes detected. The EBV status was further confirmed by Epstein-Barr encoding region (EBER) in situ hybridization (Figure 2). The second sample was a plasmablastic lymphoma (PBL) from an HIV+ patient where EBV was also detected by the LLMDA. The results demonstrated that FFPE samples could be used to reliably detect DNA sequences of known microbial origin and could therefore also be used to study microbial pathogen associations with lymphomas or other tumors in general, opening a new potential discovery tool for detection of previously unrecognized or novel associations linking pathogens with oncogenesis. Additionally, this technique can be used to assess for the persistence of known viral pathogens in tissue samples from patients who have undergone treatment to predict residual disease (See Figure 3) (See Table 2).

**Table 1:** **Distribution of EBV-status of lymphoma cases in the study**

**Type of tissue**	**EBER positive/total**
Benign lymphoid tissues	0/8
B-cell lymphomas	5/30
T-cell lymphomas	1/2
NK/T cell lymphomas	2/2
PTLDs	5/10
Hodgkin Lymphoma	1/6

**Table 2:** **Analysis of post-transplant lymphoproliferative disorder (PTLD) cases using the LLMDA**

**Sample #**	**Tumor type**	**EBV detected by LLMDA**	**Log odds score**	**In situ hybridization results**
**1**	Diffuse large B-cell lymphoma type PTLD, neck mass	Yes	121.6	EBER+
**2**	Diffuse large B-cell lymphoma PTLD, liver	Yes	114.7	EBER+
**3**	Diffuse large B-cell lymphoma type PTLD, spleen	Yes	112.4	EBER+
**4**	Classical Hodgkin lymphoma, mixed cellularity type PTLD, neck lymph node	Yes	104.4	EBER+
**5**	Extranodal NK/T cell lymphoma type PTLD, nasal mass	Yes	87.1	EBER+
**6**	Burkitt lymphoma type PTLD, neck lymph node	No	NA	EBER-
**7**	Diffuse large B-cell lymphoma type PTLD, brain mass	No	NA	EBER-
**8**	Classical Hodgkin lymphoma, nodular sclerosis type PTLD, axillary lymph node	No	NA	EBER-
**9**	Diffuse large B-cell lymphoma type PTLD, Inguinal lymph node	No	NA	EBER-
**10**	Burkitt lymphoma type PTLD, neck mass	No	NA	EBER-

The LLMDA accurately detected the presence or absence of EBV in 5 EBV-positive cases and 5-EBV-negative cases.

### EBV screening of PTLD cases

PTLD is a heterogeneous group of disorders that develop due to immunosuppressant regimens required for solid organ transplant or bone marrow allograft [9]. The risk for PTLD varies substantially depending on age, transplant type, immunosuppressant therapy, and EBV status. PTLDs are usually but not invariably associated with EBV. EBV can only be detected in half of PTLD cases that develop within a year of transplant [9,11,24]. The reported incidence, however, of EBV-negative PTLDs varies widely, and it is uncertain whether they should be considered analogous to EBV-positive PTLDs and whether they have any distinctive features [8]. We evaluated the accuracy of LLMDA for detecting EBV in 10 PTLD cases (Figure 3). DNA was isolated from the 10 frozen samples and applied to the LLMDA. Our system accurately detected the presence EBV in all the 5 EBV-positive cases which were confirmed by EBER in situ hybridization and PCR analysis (data not shown), while no EBV sequence was detected in all the 5-EBV-negative cases. These results show that the LLMDA is capable of screening EBV-associated PTLD cases.

### Detection of EBV from different lymphoma types

Out of the 58 cases of lymphoid tissues analyzed by the LLMDA, 5 out of the 30 B-cell lymphoma were positive for EBV, all 5 were from aggressive types (21 aggressive types vs 9 indolent types tested). All 9 of the indolent B-cell lymphomas were negative for EBV by LLMDA. One of two T-cell lymphoma samples (angioimmunoblastic T-cell lymphoma) was positive for EBV. Two out of two NK/T cell lymphoma samples were positive for EBV. Eight out of the eight specimens of control benign lymphoid tissues were tested negative for EBV by LLMDA. Additionally, only one of six classical Hodgkin lymphoma cases was detected for EBV by LLMDA (Table 1).

## Discussion

The link between pathogen infections and tumorigenesis has been established in many cancers [2]. Virus-associated cancers are on the rise worldwide due primarily to the aging population as well as to the increasing use of solid organ transplantation and the associated use of immunosuppressant drug regimens [7]. The identification of virus-associated malignancies will provide researchers and physicians with novel and effective therapeutic targets and treatment options. There is a need to be able to accurately and cost effectively screen increasing numbers of tumors in order to identify their cause and develop effective prevention and treatment options. The LLMDA is less expensive than sequencing per sample and provides far greater flexibility than current sequencing and PCR diagnostic techniques [23]. Although the current study focuses on EBV-associated cancers, the other viruses are also detectable when present providing a “one test detects all” technique.

Studying pathogen-induced tumorigenesis will require access to large sample pools [7]. Therefore, studies using frozen tissues come with limitations. Frozen tissues are often in short supply, and it is difficult to obtain specific tissues in significant numbers and in a timely fashion, as researchers need them. On the other hand, FFPE tissues are usually available in substantial numbers in many tissue banks. We have confirmed the ability of the LLMDA system to accurately detect EBV in FFPE samples. The option to use FFPE provides researchers with the opportunity to screen large collections of relevant tumor samples in archives worldwide, which is likely to reveal previously unknown pathogen-associated malignancies. The technique will also permit screening of samples from patients living in geographic locations from which collection and proper shipping of frozen tissues are prohibitive. The use of FFPE samples could hasten the identification of pathogen-induced malignancies and potentially help the development of therapies for these diseases.

We have previously demonstrated that the array detected viruses from a variety of human clinical samples such as urine, feces, serum, skin lesion, cerebral spinal fluid (CSF), tracheal aspirate, etc [18,19,21,25]. For example, the microarray detected BK polyomavirus positive urine samples containing ≥1000 copies/mL (or an equivalent of 5 viral copies in a Phi29-amplification reaction) [18]. In another study, the array was used to detect Kaposi-sarcoma associated herpes virus, or human herpes virus 8 from bladder cancer samples. This further demonstrated the potential of the microbial detection array technology to identify pathogens that might be linked to cancer and other diseases. The array detected viral DNA from as little as 20 fg or 100 genome copies input when combined with whole genome amplification [18,20]. Therefore, this microarray technique can be potentially used to detect EBV infection in clinical samples including blood and cerebral spinal fluid (CSF) [18-20,26].

Reliance on a technique as a clinical diagnostic tool requires that its accuracy is tested and confirmed at a reliable level. In our study, the accuracy of the LLMDA has been demonstrated by correctly identifying EBV in 5 out of 10 PTLD clinically analyzed samples. Once the EBV’s initial lytic infection is brought under control, it can persist in latency. The EBV genome circularizes, resides in the cell nucleus as an episome, and is copied by cellular DNA polymerase. Each of the EBV latency programs leads to the production of a limited, distinct set of viral proteins and viral RNAs [11]. For clinical diagnosis, EBER in situ hybridization is the standard methodology of choice for the detection of the EBV in tissue sections [16]. The large numbers of copies of EBERs can be detected in latently infected cells. Positive studies show staining in the nuclei of the EBV-infected cells. False-negative results by EBER in situ hybridization are most often due to RNA degradation which still can be detected through the LLMDA with advantage of multiple probes. The LLMDA also possesses features unavailable to PCR and sequencing as it can more easily detect multiple pathogens in the same sample and this array also permits discovery of novel pathogens. In addition, we find that EBV-infection was detected from aggressive high grade lymphomas, but not the indolent low grade lymphomas. These results support the use of this system as a reliable clinical test for a variety of other tumor types.

## Conclusions

In the present study, we were able to successfully screen FFPE lymphoma clinical cases, which will permit screening of a large volume of relevant samples, found in the archives. We demonstrated the accuracy of the LLMDA system by detecting the presence or absence of EBV in PTLD cases by screening of clinical samples. Finally, we were able to show that the system can replicate clinical results by properly identifying EBV’s association with possible progression from indolent to aggressive tumor stages. Taken together, our results support the use of the LLMDA as an important clinical tool in the screening of pathogen-associated lymphomas. In addition, this novel method has the ability to detect multiple broad-spectrum pathogen pathogens simultaneously [18].

In conclusion, using the LLMDA, we have developed an accurate and sensitive method for screening frozen and FFPE lymphoma samples. The technique performs well in the testing of most cancer types; however PCR and sequencing assays may be required to confirm results produced by the LLMDA for tumors containing only a small percentage of transformed cells infected with pathogen. Nevertheless, the LLMDA system may provide a less expensive and/or more flexible clinical alternative to current techniques with FISH, PCR analysis and sequencing.

## Methods

### Patients and tissue specimens

A total of 58 patients (36 men and 22 women) diagnosed with lymphoma were included in the study. The tissues were collected at the Biorepository tissue bank, Department of Pathology, of University of California, Davis Medical Center during January 2010 to May 2014. The 10 PTLD samples were collected in Loma Linda University Medical Center. These cases previously had been diagnosed as lymphoma, and subclassified using standard morphologic criteria, immunophenotyping, cytogenetic and selected fluorescence in situ hybridization (FISH) studies. Part of the fresh tissue samples was stored in liquid nitrogen until the extraction of DNA. The remaining tissues were fixed in formalin overnight. The study was approved by the IRB Ethics Committee of UC Davis medical center and informed consent was obtained.

### DNA extraction

DNA was isolated from FFPE tissues using the AllPrep DNA/RNA FFPE Kit (Qiagen) following the manufacturer’s protocol with minor modifications. DNA was extracted from 1, 2 or 4, 10 μm sections of 150 mm^2^ surface area.

### Microarray analysis

Genomic DNA (gDNA) was isolated from frozen and FFPE samples as described above. The gDNA was quantified using a Qubit 2.0 Fluorometer (Life Technologies). Then, 1 μg gDNA was fluorescently labeled with Cy3-labeled random nonamers using the NimbleGen One-Color Labeling Kit (Roche). Labeled gDNA was re-quantitated with the Qubit 2.0 Fluorometer and 2 μg of the labeled gDNA was hybridized to the 12×135K format of the LLMDA v.5 for 60 hr. The arrays were washed using a NimbleGen Wash Kit (Roche) and scanned at 532 nm (Cy3 channel) using the NimbleGen MS 200 Microarray Scanner (Roche). The array was analyzed using the Composite Likelihood Maximization method at the 99% threshold (signal intensity of target probes were 99% above random control probes) [17,25].

### EBER in situ hybridization for Epstein-Barr virus

All cases were subjected to in situ hybridization using oligonucleotides complementary to Epstein–Barr early RNA (EBER) transcripts in paraffin embedded tissues in an automated stainer (Integrated Oncology). The detail testing protocols using EBER riboprobes has been published [16].

## Abbreviations

EBV: Epstein-Barr virus
HHV-4: Human herpesvirus 4
LLMDA: Lawrence livermore microbial detection array
PTLD: Post-transplant lymphoproliferative disorder
EBER: Epstein-Barr virus-encoded small RNA
HPV: Human papilloma virus
KSHV: Kaposi’s sarcoma-associated herpesvirus
FISH: Fluorescence in situ hybridization
PBL: Plasmablastic lymphoma
FFPE: Formalin fixed paraffin embedded
CSF: Cerebral spinal fluid
